# Intranasal Galanin(1–15) influences depression- and anxiety-related behaviors through sex-dependent mechanisms in rats

**DOI:** 10.1186/s13293-026-00901-0

**Published:** 2026-04-25

**Authors:** Noelia Cantero-García, Antonio Flores-Burgess, Marta Flores-Gómez, Juan Pedro Pineda-Gómez, Carmelo Millón, Zaida Díaz-Cabiale

**Affiliations:** Universidad de Málaga, Instituto de Investigación Biomédica de Málaga, Facultad de Medicina, Campus de Teatinos s/n, Málaga, 29071 Spain

**Keywords:** Galanin, Intranasal administration, Sex differences, Depression, Rats

## Abstract

**Background:**

Major depressive disorder affects more women than men, possibly due to sex-specific biological and environmental factors. Previous studies have shown that Galanin, as well as specific fragments of it, such as Galanin 1–15 [GAL(1–15)], play a crucial role in modulating depression in animal models by acting on brain regions like the hippocampus and dorsal raphe nuclei. Until now, its effects had only been studied in male rats, where GAL(1–15) produces depressive and anxiogenic behaviors in behavioral tests. This study analyzes, for the first time, the effects of GAL(1–15) on female rats and compares the expression of galaninergic and serotonergic genes between males and females to understand sex-dependent mechanisms in depression.

**Methods:**

For this purpose, the effect of GAL(1–15) administered intranasally in female rats was assessed, using validated behavioral tests for depression: the Forced Swim Test (FST) and the Tail Suspension Test (TST), as well as the anxiety behavior test: the Open Field Test (OFT). Furthermore, the expression of galaninergic genes (GAL, GALR1, GALR2, GALR3) and the 5-HT1A receptor was compared in untreated male and female rats using qPCR in key brain regions implicated in depressive disorder: the dorsal raphe nucleus (DR), the dorsal hippocampus, and the prefrontal cortex (PFC).

**Results:**

We demonstrated that GAL(1–15) intranasally administered in female rats strongly produced depressive behavior in the FST as well as in the TST. Moreover, in the anxiety test, intranasal GAL(1–15) at high doses produced anxiety behavior in the OFT. In addition, the qPCR study revealed that naïve female rats exhibited increased expression of the galaninergic system and 5-HT1A receptor compared to naïve male rats in the DR, dorsal hippocampus, and PFC, several nuclei implicated in depression.

**Conclusions:**

These results demonstrate the existence of sexual dimorphism in the galangergic system.These findings on sex-specific neurobiological variations are crucial for advancing toward more precise therapies tailored to the specific characteristics of each sex in the treatment of depression.

**Supplementary Information:**

The online version contains supplementary material available at 10.1186/s13293-026-00901-0.

## Abstract (plain language summary)

Major depressive disorder is a mental disorder that affects more women than men, possibly due to multiple causes, including genetic, contextual, and sex-specific biological factors. Previous studies in animal models have shown that Galanin, a peptide active in the Central Nervous System (CNS) and Galanin fragments, such as Galanin 1–15 [GAL(1–15)], play a key role in depression-related behaviors and act on brain regions crucial to this disorder, such as the hippocampus, responsible for memory and regulation, and dorsal raphe nuclei, with an important role in mood disorders. Until now, the effects of GAL(1–15) had only been studied in male rats, where GAL(1–15) produces behaviors related to depression and anxiety. In this study, we examined GAL(1–15) administered intranasally in female rats in tests related to depression and anxiety. In addition, we compared specific biological brain signals—genes related to the galanin and serotonin systems—between males and females without previous treatment. The results show that high doses of intranasal GAL(1–15) produced depressive and anxiogenic effects in female rats in several behavioral tests. Furthermore, the molecular study revealed that female rats exhibited higher expression of receptors related to the effects of GAL(1–15) (galaninergic system) and higher expression of receptors linked to mood (serotonergic system). These results demonstrated that biological differences exist between males and females. These findings are crucial for advancing towards more precise treatments tailored to the characteristics of each sex in the treatment of depression.

**Supplementary Information:**

The online version contains supplementary material available at 10.1186/s13293-026-00901-0.

## Highlights

Intranasal administration of GAL(1-15) in female rats induces depressive- and anxiety-like behaviors.

In female rats, relatively high intranasal doses of GAL(1-15) are required to produce these behavioral effects.

Female rats show increased galaninergic and serotonergic system gene expression in the dorsal raphe nuclei, dorsal hippocampus, and prefrontal cortex compared to male rats.

These results suggest the existence of sexual dimorphism in the galangergic system.

**Supplementary Information:**

The online version contains supplementary material available at 10.1186/s13293-026-00901-0.

## Introduction

Major depressive disorder (MDD) is a highly prevalent and debilitating condition characterized by persistent low mood, anhedonia, and, in severe cases, suicidal behavior. It disproportionately affects women, who are nearly twice as likely as men to be diagnosed—21% compared to 12%—and to experience recurrent episodes [1, 2]. This disparity in prevalence between sexes can be attributed, at least in part, to environmental risk factors and sex-specific biological influences. Understanding these factors, along with studying the neurophysiological basis of depression, is essential for gaining deeper insight into how individual, sex, and gender differences shape the presentation and progression of MDD [2]. Despite the apparent sex differences in MDD, the majority of preclinical research has historically relied on male animal models, potentially neglecting critical sex-specific mechanisms that influence both vulnerability to depression and responses to treatment [3, 4].

Galanin (GAL) is a neuropeptide widely expressed in neurons within the central nervous system (CNS), and it is involved in various physiological processes and diseases in animal models, including mood regulation and depression [5–7], through its three GAL receptors (GALR) [8]. GALR1 and GALR3 activation is linked to depressive-like behavior, while GALR2 stimulation has anti-depressant-like effects [9–11]. RNA blot analysis showed higher GAL gene expression in the anterior pituitary of female rats compared to males [12]. Treatment with 17β-estradiol increases GAL mRNA levels in the anterior pituitary and hypothalamus, contributing to elevated GAL in females [13]. Additionally, GAL microinjection into the posterior amygdala enhances sexual behavior in testosterone-treated male rats, highlighting the interaction between GAL and sex hormones in regulating behavior [13].

There are GAL-derived fragments that also exhibit biological activity in the CNS, such as the Galanin (1–15) [GAL(1–15)]. GAL(1–15) is also active in mood disorders, acting through GALR1-GALR2 heteroreceptor complexes, particularly in the dorsal hippocampus and dorsal raphe (DR), resulting in depressive-like effects and anxiogenic-like effects in male rats [7, 14, 15]. Interestingly, GAL(1–15) enhanced the antidepressant effects induced by the 5-HT1AR agonist 8-OH-DPAT in the forced swimming test (FST) [16] involving alterations in the binding characteristics and mRNA levels of 5-HT1AR in the dorsal hippocampus and DR. Building on these findings, GAL(1–15) has shown promise as an adjunct to selective serotonin reuptake inhibitors (SSRIs). In male rats, GAL(1–15) enhanced fluoxetine (FLX)’s antidepressant effects and reversed FLX-induced memory impairment through 5-HT1A receptor modulation in the hippocampus and the prefrontal cortex (PFC) [17–19]. In olfactory bulbectomy male rats, GAL(1–15) potentiated SSRI effects on despair and anhedonia by modulating 5-HT1A receptors at membrane and transcriptional levels in the hippocampus [19–21]. Additionally, GAL(1–15) enhanced FLX’s antidepressant-like effects in the Wistar-Kyoto rat model [22].

Recently, we have described that GAL(1–15) administered intranasally produces depressive-like effects in male rats, increasing immobility and decreasing swimming time in the FST, consistent with previous intracerebroventricular findings [7, 23]. The intranasal route induced dose-dependent behavioral effects, with the highest dose causing sustained mood modulation lasting at least an hour post-administration. Intranasal administration of GAL(1–15) effectively replicates the effects of intracerebroventricular delivery, offering a non-invasive route that bypasses the blood-brain barrier [23]. This method presents a promising and clinically feasible strategy for sustained modulation of mood-related behaviors, supporting its potential use in human treatment. Furthermore, GAL(1–15) has a favourable safety profile, making it suitable for development as a therapeutic candidate [23]. These evaluations demonstrated good tolerability and no significant adverse effects, indicating its promising potential for further pharmacological development in the context of depressive and mood disorders.

Research on GAL(1–15)-mediated effects has focused exclusively on male rats, leaving a critical gap in understanding its role in females. To address this gap, we aim to investigate the impact of GAL(1–15) in female rats to understand better sex-specific mechanisms involved in depression and to inform the development of more targeted and effective therapeutic strategies.

In this study, we will characterize for the first time the behavioral response to intranasally administered GAL(1–15) in female rats using validated tests of depressive- and anxiety-like behavior. In addition, we compared the expression of genes related to the galaninergic system (GAL, GALR1, GALR2, GALR3) and the serotonergic system (5-HT1A receptor) in naive male and female rats in key brain regions involved in depressive disorder: DR, hippocampus, and PFC.

## Materials and methods

### Animals

Female (body weight 175–200 g) and male Sprague-Dawley rats (body weight 200–225 g) were obtained from Charles River (Barcelona, ​​Spain) and maintained in a humidity-controlled and temperature-controlled (20–22 °C) room on a 12-hour light/dark cycle and fed ad libitum. Male and female rats were housed individually in standard cages and maintained separately by sex. They had a period of adaptation and handling before the performance of the behavioral tests. All animal experimentation was conducted in accordance with the University of Málaga Guidelines for the Care and Use of Laboratory Animals (Ethic Code: 22/05/2017/066).

### Administration of substances and drugs

Female rats were administered under light anaesthesia with isoflurane (3–4% for induction and 1.5–2% for maintenance) for approximately 2–3 min, sufficient to prevent movement during intranasal administration. A single intranasal infusion of GAL(1–15) (TOCRIS, Bristol, United Kingdom, molecular weight = 1557 g/mol) was administered at doses of 75 µg, 150–300 µg, equivalent to those used with other peptides [24, 25]. Doses were dissolved in 20 µl of distilled water, 10 µl into each nose with a pipette and disposable plastic tip, one hour before behavioral testing, as described previously [23–26]. Care was taken to avoid contact with the intranasal mucosa. After intranasal administration, the animal’s head was held back for approximately 15 s to prevent loss of solution from the nostrils.

The estrous cycle of female rats was monitored throughout the study, as described previously [27] to ensure they were in the same phase of the cycle on the experiment days.

### Behavioral assessment

#### Depressive behavior: Forced swimming test (FST) and Tail Suspension Test (TST)

Depressive behavior was assessed using the FST, performed as described previously [7, 16, 19, 22, 23, 28]. Briefly, two swimming sessions were conducted: a 15-minute pretest followed 24 h later by a 5-minute test. Animals were individually placed in a vertical glass cylinder of 20 cm diameter containing water (25 °C) to a height of 30 cm. The total duration of immobility, swimming, and climbing behavior was recorded during the second 5-minute test period. Behavioral scoring was conducted by a blinded experimenter using coded videos to ensure objectivity and impartiality.

For this purpose, different doses of GAL(1–15) were administered intranasally to female rats. Groups of rats received GAL(1–15) intranasally at doses of 75 µg (low dose), 300 µg (high dose), or distilled water one hour before the test.

The TST also assesses hopelessness and was performed as described previously [7, 22, 23, 28]. Briefly, rats were hung upside down using adhesive tape to fix their tails to a rope through an eyebolt at 60 cm. The animal was considered immobile when it was not making any movements, struggling, attempting to catch the adhesive tape, body torsions, or jerkstime. The total duration of immobility and mobility behavior was recorded during the second 6-minute test. Behavioral scoring was conducted by a blinded experimenter using coded videos to ensure objectivity and impartiality.

For this, groups of rats received GAL(1–15) intranasally at 300 µg or distilled water one hour before the test.

#### Open Field Test (OFT)

Anxiety behavior and locomotor activity were registered in the open field (100 × 100 × 50 cm), where animals were individually placed and allowed to explore freely. Their behavior was recorded over 5 min by a ceiling-mounted video camera, and locomotor activity was analyzed using the video-tracking software Ethovision XT (Noldus, S.L.). Behavioral scoring was conducted by a blinded experimenter using coded videos to ensure objectivity and impartiality.

After each trial, all surfaces were cleaned with a paper towel and 70% ethanol solution. Total time spent (s), number of entries into the center, distance travelled (cm) and mean speed (cm/s) were recorded. Entry into the center was defined when the center-point of the body of the rat crossed in the predefined central zone.

For this, groups of rats received GAL(1–15) intranasally at doses of 150 µg, 300 µg, or distilled water one hour before the test.

### Gene expression in the dorsal raphe, dorsal hippocampus and prefrontal cortex

A different group of male and female rats that had not received any treatment were euthanised by decapitation, and the brains were rapidly removed and frozen until use. The nuclei dissections were conducted as described [22, 29, 30] with modifications. Brains were sliced on the brain matrix (1 mm) and kept at -20 °C until each region of interest came into the cutting plane. For dorsal DR analysis, one 1 mm coronal brain section was obtained from bregma − 7.8 mm (Paxinos, 1986). For the dorsal hippocampus study, two consecutive 1 mm coronal slices were made corresponding to approximately bregma − 3.60 mm (Paxinos, 1986). For the PFC study, two consecutive 1 mm coronal slices were made, corresponding to an approximate bregma coordinate of + 3.20 mm (Paxinos, 1986). Tissues of interest were dissected using a punching device with a 2 mm internal diameter. DR, and bilateral punches of Dorsal Hippocampus and PFC were collected into Eppendorf tubes.

#### RNA isolation and quantitative real-time polymerase chain reaction (RT-PCR) analysis

The procedure to perform RNA isolation and RT-PCRs was described previously [17, 29, 30] (see the supplementary material for details).

The primer sequences used to evaluate the mRNA expression levels of the genes GAL, GALR1, GALR2, GALR3, and 5-HT1A are shown in the supplementary material.

### Statistical analysis

Data are presented as the mean ± standard error of the mean, and sample numbers (n) are indicated in figure legends. Animals were excluded from the analysis when their values exceeded ± 2 standard deviations from the group mean, according to predefined criteria applied consistently across all experimental groups. All data were analyzed using GraphPad PRISM 8.0 (GraphPad Software, San Diego, CA, USA). For comparing two experimental conditions, Student´s unpaired t-tests were performed. No formal correction for multiple comparisons was applied, as the number of planned t‑tests was limited and hypothesis‑driven. For comparing more than two groups, one-way analysis of variance (ANOVA) was performed. Fisher’s least significant difference (LSD) comparison post-test was performed to control for performing multiple t tests only when the F ratio in the one-way ANOVA was statistically significant. Differences were considered statistically significant at *p* ≤ 0.05 (*p < 0.05, **p < 0.01, ***p < 0.001).

## Results

### Intranasally GAL(1–15) induced depression-related behavior in female rats

**Fig. 1:**
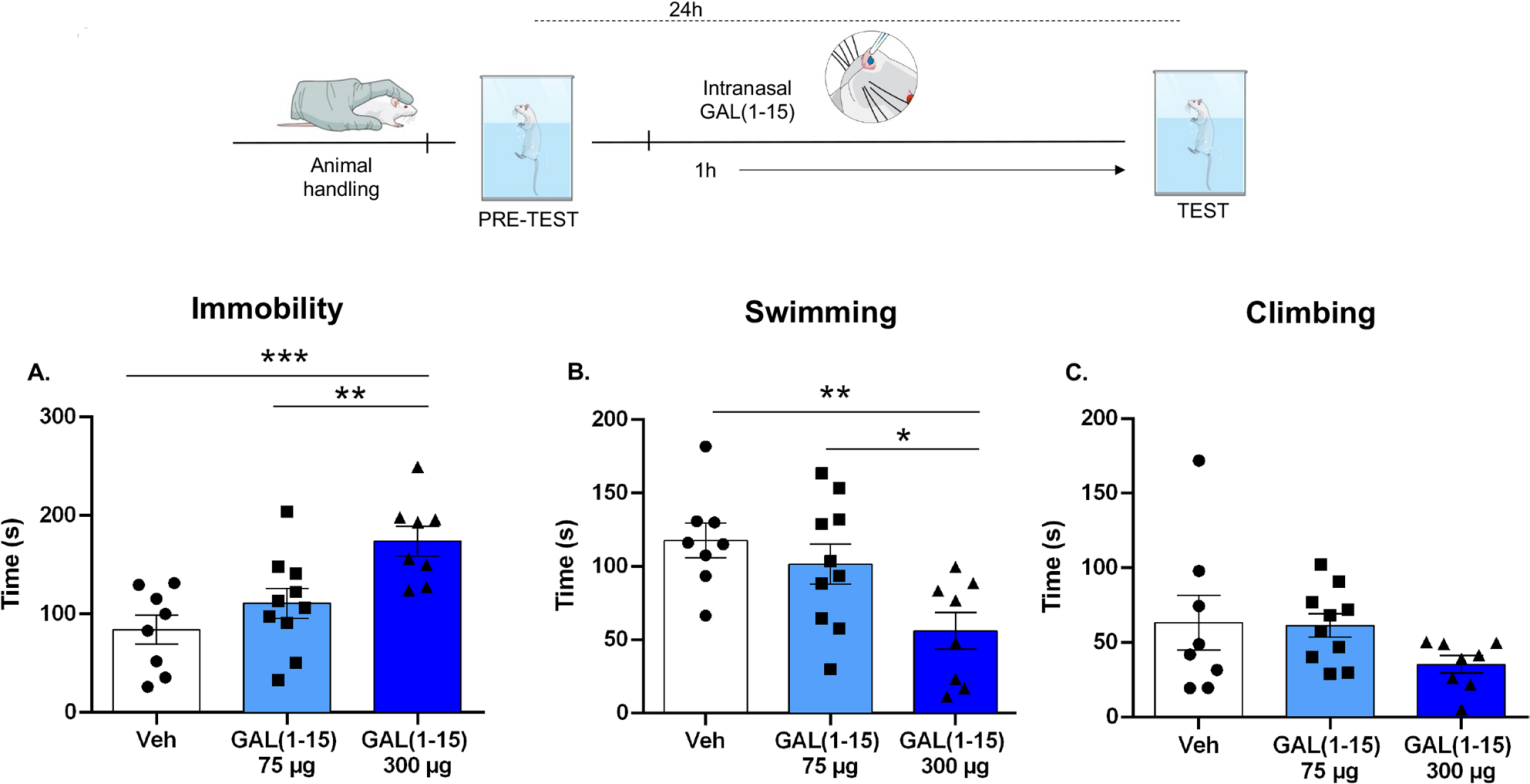
Effect of the intranasal administration of Galanin(1–15) [GAL(1–15)] in the Forced Swimming Test (FST) in female rats. GAL(1–15) 75 µg, 300 µg, or distilled water was administered intranasally one hour before the test. Rats received 10 µl of the substance into each nostril. Distilled water-injected rats were the vehicle group (*n* = 8); GAL(1–15) 75 µg group (*n* = 10); GAL(1–15) 300 µg group (*n* = 8). **A, B, C**: Vertical bars represent the mean ± standard error of the mean immobility, swimming and climbing time. * *p* < 0.05 vs. GAL(1–15) 75 µg group, ** *p* < 0.01 vs. the rest of the groups; *** *p* < 0.001 vs. vehicle group according to a one-way analysis of variance (ANOVA) between the experimental groups

In the FST, two doses of GAL(1–15) intranasally were tested in female rats. We observed that the administration of GAL(1–15) (300 µg) induced a strong increase by 106.8% in the immobility time (one-way ANOVA F_2,23_=8.54, *p* = 0.002; Fisher´s LSD post hoc: *p* < 0.001; Fig. 1A) and decreased by 52.4% in swimming time (one-way ANOVA F_2,23_=5.69, *p* = 0.009; Fisher´s LSD post hoc: *p* < 0.01; Fig. 1B) compared with the vehicle group.

However, GAL(1–15) at 75 µg lacked an effect in the FST, suggesting that a higher dose of GAL(1–15) is needed to produce depressive-behavior in female rats.

**Fig. 2:**
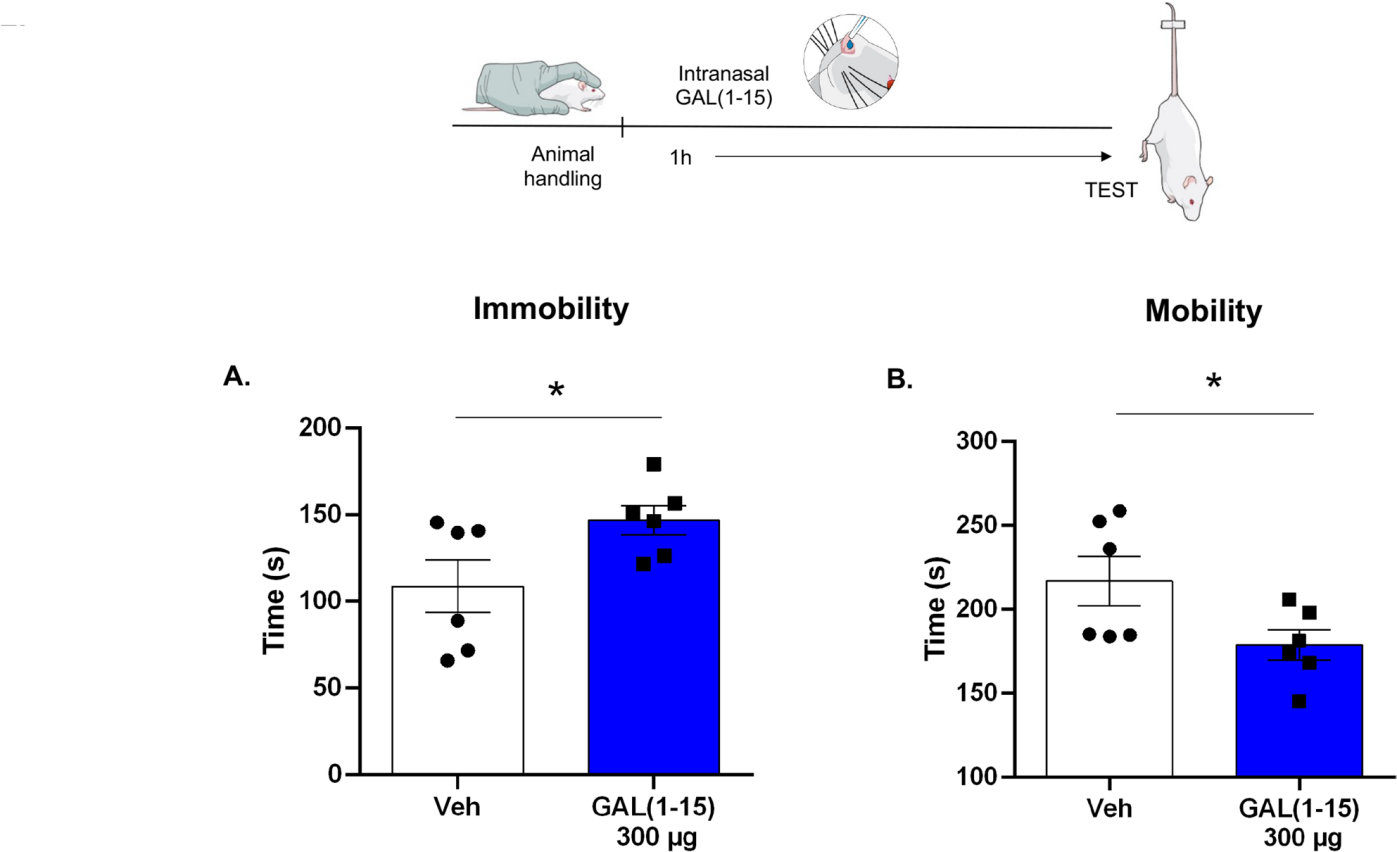
Effect of the intranasal administration of GAL(1–15) in the Tail Suspension Test (TST) in female rats. GAL(1–15) 300 µg or distilled water was administered intranasally one hour before the test. Rats received 10 µl of the substance into each nostril. Distilled water-injected rats were the vehicle group (*n* = 6); GAL(1–15) 300 µg group (*n* = 6). **A, B**: Vertical bars represent the mean ± standard error of the mean immobility and mobility time. * *p* < 0.05 vs. the vehicle group according to a Student t-test

In climbing time, no effect was observed for any of the tested doses (Fig. 2C).

In addition, intranasal administration of GAL(1–15) was evaluated in the TST. The results showed that the effective dose (300 µg) induced a significant increase in immobility time (Student t-test, t_10_= 2.19, *p* < 0.05; Fig. 2A) and a decrease in mobility time (Student t-test, t_10_= 2.20, *p* < 0.05; Fig. 2B), compared to the vehicle group, thus highlighting a notable effect of this dose on helplessness behavior in female rats.

### Intranasal administration of GAL(1–15) produces anxiogenic effects in female rats

**Fig. 3:**
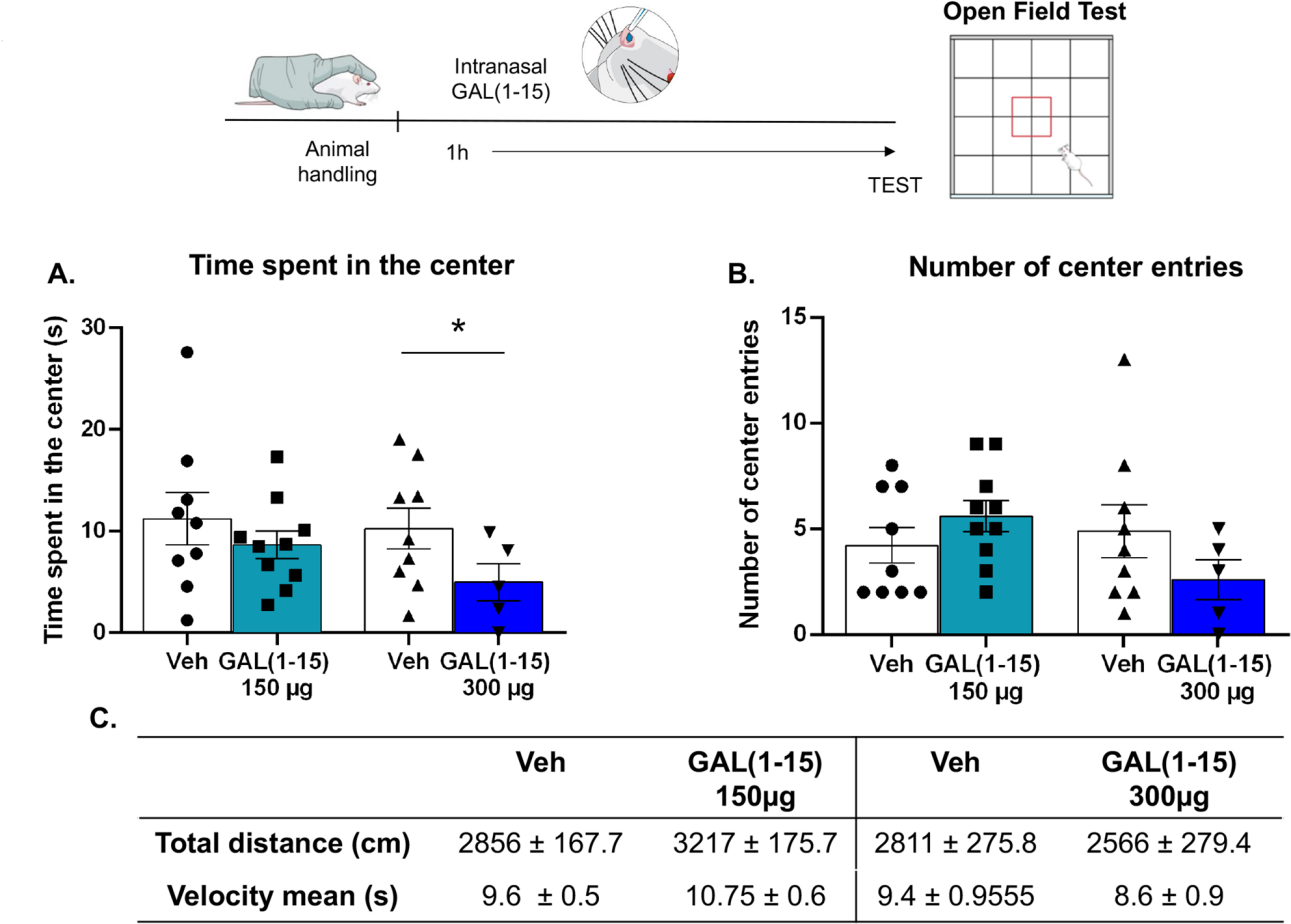
Effect of the intranasal administration of Galanin(1–15) in the Open Field Test (OFT) in female rats. GAL(1–15) 150 µg (*n* = 10), 300 µg (*n* = 5) or distilled water (*n* = 9) was administered intranasally one hour before the test. Rats received 10 µl of the substance into each nostril. Distilled water-injected rats were the vehicle group. **A, B**: Vertical bars represent the mean time spent in the center and the number of entries into the center. * *p* ≤ 0.05 vs. vehicle group according to a Student t-test. **C.** Data represent ± the mean of the total distance and velocity mean. According to a Student t-test, there were no differences between the experimental groups

The anxiety state was evaluated in the female rats by performing the OFT. The effects of GAL(1–15) intranasally were tested at doses of 150 µg and 300 µg. The dose of 300 µg induced a notable reduction, 51.4%, in the time spent in the central area of ​​the arena (Student t-test, t_12_ = 1.76, *p* ≤ 0.05; Fig. 3A) and a similar, but not statistically significant, reduction in the number of entries in the central zone (Fig. 3B). In contrast, the 150 µg dose did not affect either parameter.

Importantly, assessments of locomotor activity revealed no changes at either dose, indicating that the observed behavioral effects were not due to alterations in general motor function (Fig. 3C). These results suggest that the higher dose of GAL(1–15) selectively influences anxiety-like behavior without impairing locomotor activity.

### Sex differences in the galaninergic system in male and female rats

The expression of different genes of the galaninergic system and the 5-HT1A receptor was compared in male and female rats in the DR, dorsal hippocampus and PFC.

**Fig. 4:**
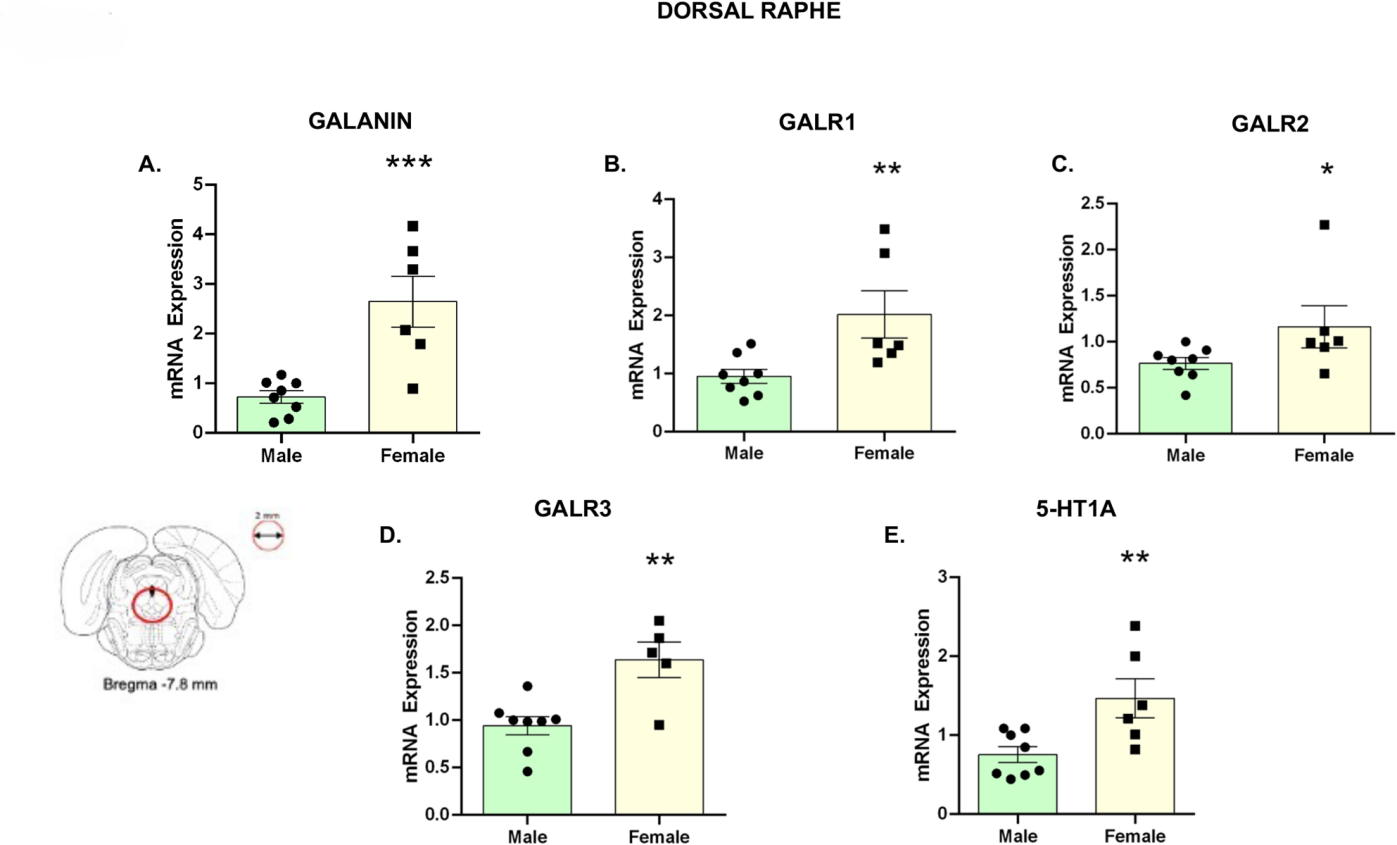
Comparison of expression levels in the galaninergic system in males and females in the dorsal raphe. Naive male (*n* = 8) and female rats (*n* = 5–6) were euthanized by decapitation for a qPCR study. **A, B, C, D, E**: Vertical bars represent the mean ± standard error of the mean of Galanin, GALR1, GALR2, GALR3, and 5HT1A mRNA expression. * *p* < 0.05, ** *p* < 0.01; *** *p* < 0.001 vs. male rats group according to a Student t-test

In the DR, female rats showed higher mRNA expression of the GAL receptor (Student t-test, t_12_= 4.17, *p* < 0.001; Fig. 4A) and also a significant increase in the GALR1 (Student t-test, t_12_= 2.85, *p* < 0.01; Fig. 4B), GALR2 (Student t-test, t_12_= 1.89, *p* < 0.05; Fig. 4C), and GALR3 (Student t-test, t_11_= 3.66, *p* < 0.01; Fig. 4D) mRNA expression, compared with the male rats. In addition, 5HT1A was also increased in female rats compared with male rats (Student t-test, t_12_= 2.96, *p* < 0.01; Fig. 4E) in DR.

**Fig. 5:**
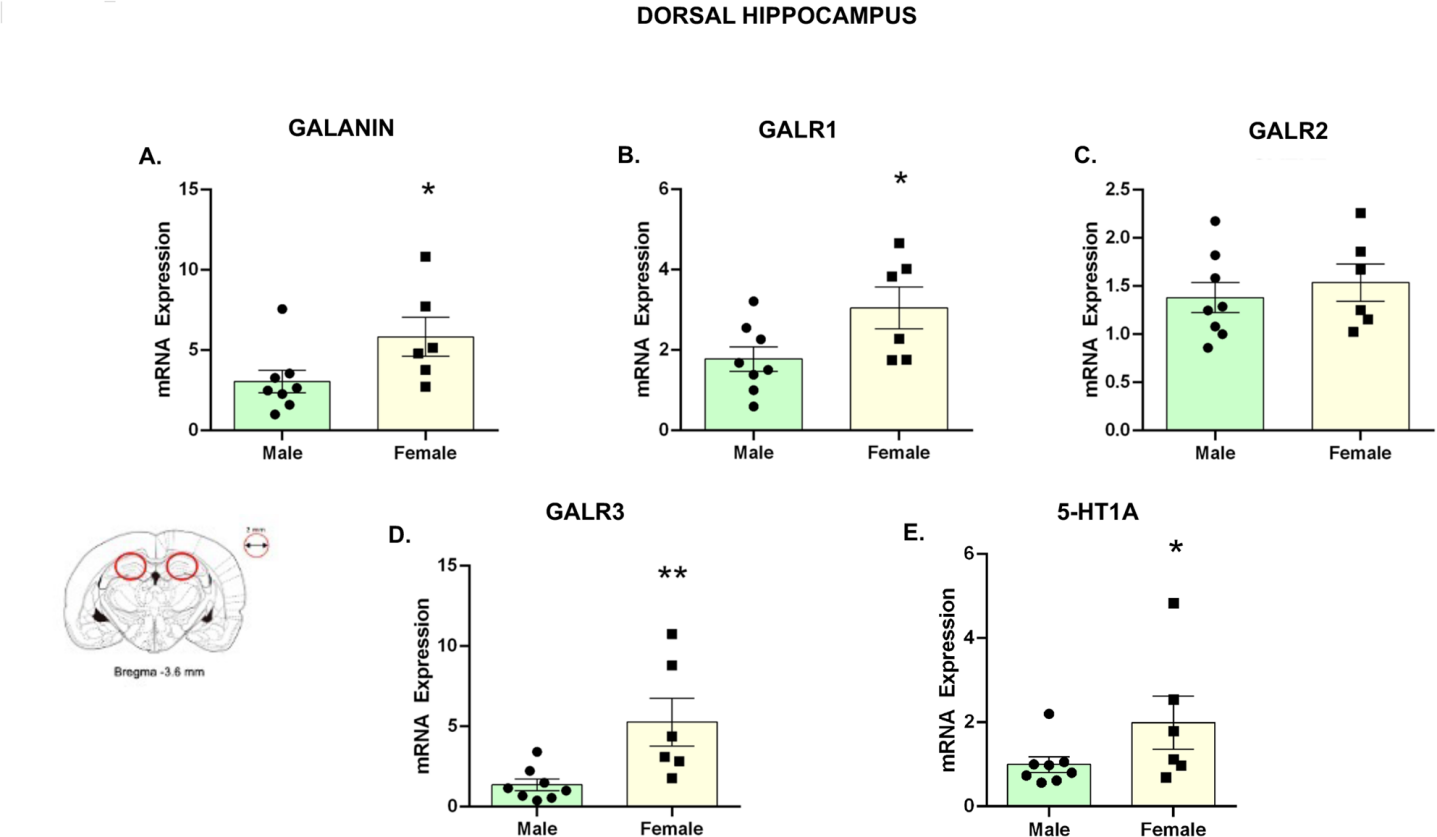
Comparison of expression levels in the galaninergic system in males and females in the dorsal hippocampus. Naive male (*n* = 8) and female rats (*n* = 6) were euthanized by decapitation for a qPCR study. **A, B, C, D, E**: Vertical bars represent the mean ± standard error of the mean of Galanin, GALR1, GALR2, GALR3, and 5HT1A mRNA expression. * *p* ≤ 0.05, ** *p* < 0.01 vs. male rats group according to a Student t-test

The genes of the Galaninergic system were also studied in the dorsal hippocampal nucleus, observing that in female rats, there was a significant increase in the mRNA expression of the galaninergic gene (Student t-test, t_12_= 2.10, *p* < 0.05; Fig. 5A), GALR1 (Student t-test, t_12_= 2.25, *p* < 0.05; Fig. 5B), and GALR3 (Student t-test, t_12_= 2.92, *p* < 0.01; Fig. 5D) compared to untreated male rats. The 5-HT1A receptor showed an increase in its expression in female rats (Student t-test, t_12_= 1.72, *p* ≤ 0.05; Fig. 5E) compared to males, while no significant differences were observed in the GALR2 receptor.

**Fig. 6:**
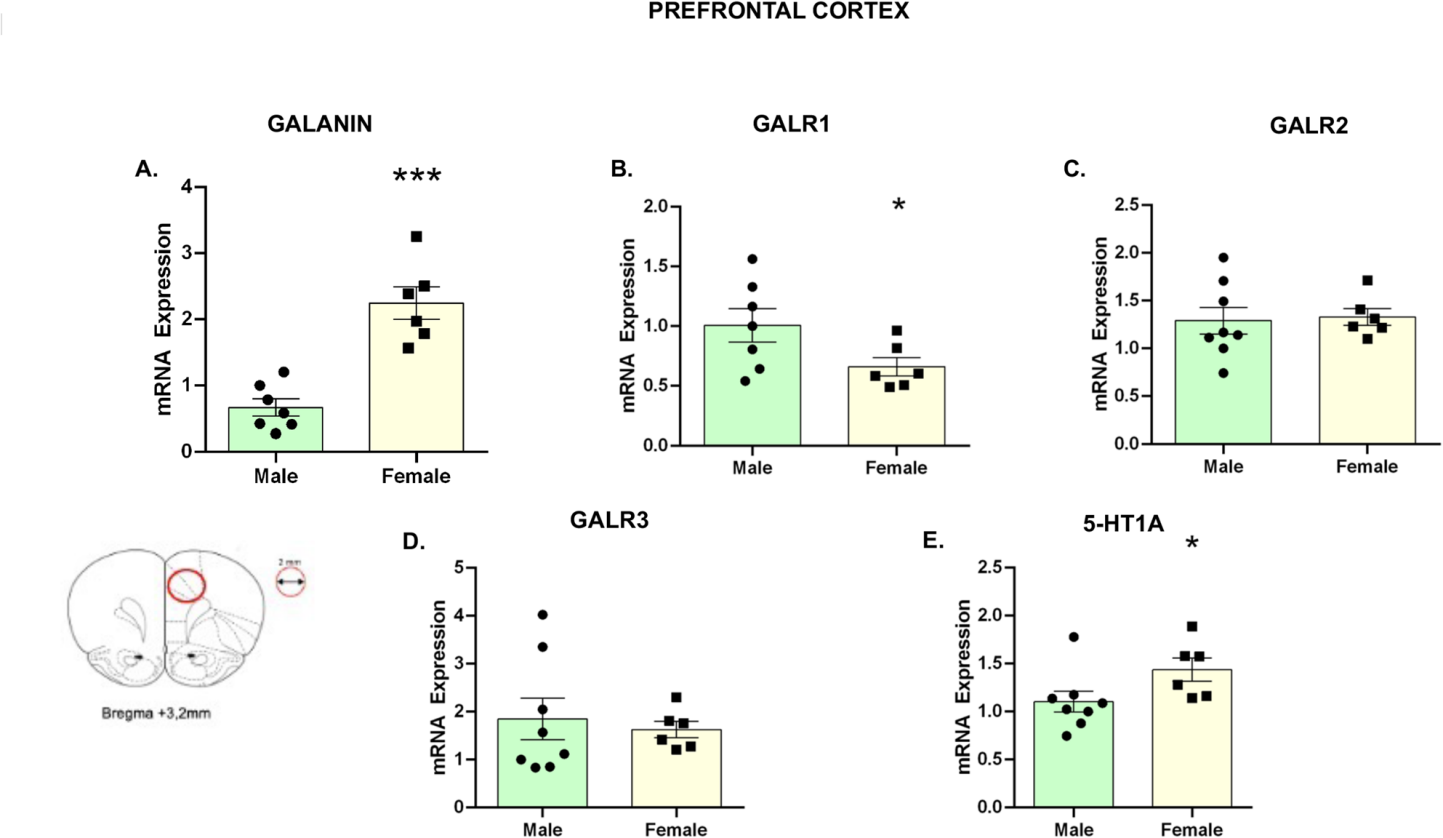
Comparison of expression levels in the galaninergic system in males and females in the prefrontal cortex. Naive male (*n* = 7–8) and female rats (*n* = 6) were euthanized by decapitation for a qPCR study. **A, B, C, D, E**: Vertical bars represent the mean ± standard error of the mean of Galanin, GALR1, GALR2, GALR3, and 5HT1A mRNA expression. * *p* < 0.05, *** *p* < 0.001 vs. male rats group according to a Student t-test

In the PFC, female rats showed a significantly increased mRNA expression of GAL (Student t-test, t_11_= 5.89, *p* < 0.001; Fig. 6A) and in 5HT1A (Student t-test, t_12_= 2.05, *p* < 0.05; Fig. 6E) receptors compared with male rats. However, it was observed that the expression of the GALR1 receptor in female rats was significantly decreased compared to male rats (Student t-test, t_11_= 2.05, *p* < 0.05; Fig. 6B). No significant differences were observed in the GALR2 and GALR3 receptor.

## Discussion

In the present study, we demonstrated that GAL(1–15) administered intranasally in female rats produced depressive behavior in both the FST and the TST. Moreover, in the anxiety test, intranasal GAL(1–15) at high doses produced anxiety behavior in the OFT. Notably, female rats exhibited increased expression of the Galaninergic system and 5-HT1A receptor compared with male rats in several nuclei implicated in depression. These results show the existence of sexual dimorphism in the galaninergic system.

The effects of intranasal administration of GAL(1–15) on depression-associated behaviors in female rats were assessed using the FST and the TST, well-established paradigms for characterizing depressive phenotypes and evaluating antidepressant efficacy in rodent models [7, 19, 22, 23, 29]. In the present study, we report for the first time that GAL(1–15) induces depressive-like behavior in female rats, as demonstrated by increased immobility in both the FST and TST. Notably, these effects were achieved for at least one hour following the administration of GAL(1–15), suggesting a sustained impact on mood-related behavior. In addition to its depressive-like effects, GAL(1–15) also influenced anxiety-related behaviors in females.

Previous studies by our group have established that GAL(1–15) elicits strong depressive-like and anxiogenic-like responses in male rats. Specifically, intracerebroventricular administration of GAL(1–15) significantly increased immobility and reduced swimming behavior in the FST, and also an increase in immobility time in the TST, consistent with a robust depressive-like phenotype [7]. Furthermore, GAL(1–15) produced marked anxiogenic-like effects in the OFT and in the Dark/Light Test [7]. Together, our findings extend previous observations in males to females, underscoring the critical role of GAL(1–15) in modulating both depressive and anxiety-related behaviors across sexes.

Regarding the administration pathway, the intranasal route offers a non-invasive and efficient method for delivering compounds directly to the central nervous system. This mechanism enables rapid distribution across multiple brain regions, thereby enhancing the translational relevance of targeting neuropeptides within the brain [31–34].

Recent studies in male rats have shown that intranasal administration of GAL(1–15) induces depressive-like behavior, characterized by increased immobility and reduced swimming time in the FST, with the 75 µg dose eliciting the most consistent and lasting effects [23]. In the present study, we replicated these depressive-like effects in female rats; however, a higher dose of 300 µg was required to produce comparable behavioral changes, while the 75 µg dose had no significant effect. These findings suggest a sex-dependent difference in sensitivity to GAL(1–15), potentially reflecting underlying neurobiological mechanisms influencing galanin signalling pathways.

We investigated the galaninergic system in treatment-naive male and female rats by examining RNA expression of galanin receptors and related serotonergic markers in central brain regions implicated in depression—the DR, dorsal hippocampus, and PFC [35–37]. Female rats exhibited significantly higher mRNA levels of the galaninergic system and the 5-HT1A receptor across all three nuclei.

Interestingly, GALR1 mRNA expression in the PFC was reduced in females compared to males, arguing against a generalized upregulation of gene expression in female brains and supporting gene-specific sex-dependent regulation. In previous studies, we showed that the GALR1/GALR2 heteroreceptor complexes in the dorsal hippocampus and especially in the DR, were involved in depression-related and anxiogenic-like GAL(1–15) effects [7, 14]. Our data suggest that enhanced galaninergic expression may underlie the reduced sensitivity to GAL(1–15) observed behaviorally in females, since more ligand is required to activate a greater number of GALR1/GALR2 complexes in females. This molecular evidence supports that sex-dependent variations in galaninergic signalling contribute to differential behavioral responses to GAL(1–15) administration in depression models.

Interestingly, the increase in 5-HT1A receptor mRNA expression in females across DR, dorsal hippocampus, and PFC suggests enhanced potential for interaction with GAL receptors. Previous studies in males have shown that GAL(1–15) enhances the antidepressant effects of the 5-HT1A receptor agonist, likely via GALR1-GALR2-5-HT1A receptor complexes [6, 16]. This increased 5-HT1A expression in females may facilitate complex formation, contributing to sex-specific modulation of the serotonergic system by GAL(1–15); however, further detailed studies are needed to confirm this.

This study reveals sexual dimorphism in the galaninergic system within key brain regions implicated in depression—the DR, dorsal hippocampus, and PFC. These differences in receptor expression and signalling may underlie the higher doses of GAL(1–15) needed to evoke depressive and anxiogenic behaviors in female rats compared to males.

The PFC dissection was focused primarily on the anterior cingulate cortex, a region critically implicated in stress and depression. Nevertheless, other prefrontal subregions, such as the prelimbic cortex, are also likely to contribute to these processes, and their examination will be important in future studies to fully characterize region-specific and sex-dependent galanin system alterations. One limitation of the present study is the exclusion of some animals whose values exceeded ± 2 standard deviations from the group mean, according to predefined criteria applied consistently across all experimental groups, which resulted in slight variability in sample sizes across experimental tests and should be considered when interpreting the findings.

The observed sex-dependent differences in galanin and 5-HT1A receptor expression, coupled with dose-dependent behavioral effects in females following intranasal administration, have important implications for depression therapeutics. These findings highlight that galaninergic modulation may produce divergent outcomes based on sex and dosage, emphasizing the need to incorporate these factors into pharmacological development. Given the higher prevalence of affective disorders in women, elucidating how differential GAL receptor expression shapes emotion regulation could enable precision interventions tailored to individual biological profiles. Future studies should validate these effects in human cohorts and test sex-specific GAL(1–15) via intranasal delivery.

In conclusion, our findings support the intranasal administration of GAL(1–15) as a safe and effective delivery method with sex-dependent dosing considerations, reflecting the inherent sexual dimorphism in the galaninergic system. Understanding these sex-specific neurobiological variations is crucial for developing targeted therapeutic strategies for depression.

## Supplementary Information

Supplementary Material 1

## Data Availability

The data that support this study are openly available in RIUMA- University of Malaga at http://doi.org, reference number [once the manuscript is accepted for publication.
