# Supplementary material for: Intranasal Galanin(1–15) influences depression- and anxiety-related behaviors through sex-dependent mechanisms in rats
Source: Biol Sex Differ. 2026 Apr 25;17:118. doi: 10.1186/s13293-026-00901-0 (PMC13261986; doi:10.1186/s13293-026-00901-0)
Supplement: Supplementary file 1 — Supplementary Material 1 [file 13293_2026_901_MOESM1_ESM.docx]

**SUPPLEMENTARY MATERIAL**

**RNA insolation and quantitative real- time polymerase chain reaction analysis**

As previously described (Cantero-García et al., 2022; Flores-Gómez et al., 2025), total RNA was isolated from the punches of DR, Dorsal Hippocampus and PFC using RNeasy Lipid Tissue Kit (Qiagen, Hilden, Germany). cDNA was obtained using a Reverse Transcriptase Core Kit (Eurogentec, Seraing, Belgium). These steps were performed according to the manufacturer’s instructions.

All PCR were conducted in triplicate using FastStart essential DNA green master (Roche Diagnostics GmbH, Mannchem, Germany) in LigtCycler 96 system (Roche Diagnostics GmbH, Mannchem, Germany). The data were analysed using the comparative Ct method and normalized to measures of glyceraldehyde- 3-phosphate dehydrogenase (GAPDH) mRNA.

The primer sequences used in this study are:

| **Gene** | **Forward primer (5´-3´)** | | **Reverse primer (5´-3´)** |
| --- | --- | --- | --- |
| **GAL** | | TTGCTAGTTTTTATTATGTCACA | AAAAGAATCTTCCGCCAAC |
| **GALR1** | | AAAACTGGACAAAACTTAGCC | GGATACCTTTGTCTTTGCTC |
| **GALR2** | | AACAGGAATCCACAGACC | CCCTTTGGTCCTTTAACAAG |
| **GALR3** | | ACAGATCTCTTCATCCTCAAC | AGATGAGCAGATGTACCG |
| **5HT1A** | | AACTATCTCATCGGCTCC | ACATCCAGGGCGATAAAC |

**Table S1. Oligonucleotide primers used in qPCR.**
